# Development and Validation of a Biomarker for Diarrhea-Predominant Irritable Bowel Syndrome in Human Subjects

**DOI:** 10.1371/journal.pone.0126438

**Published:** 2015-05-13

**Authors:** Mark Pimentel, Walter Morales, Ali Rezaie, Emily Marsh, Anthony Lembo, James Mirocha, Daniel A. Leffler, Zachary Marsh, Stacy Weitsman, Kathleen S. Chua, Gillian M. Barlow, Enoch Bortey, William Forbes, Allen Yu, Christopher Chang

**Affiliations:** 1 GI Motility Program, Cedars-Sinai Medical Center, Los Angeles, California, United States of America; 2 Beth Israel Deaconess Medical Center, Boston, Massachusetts, United States of America; 3 Department of Biostatistics, Cedars-Sinai Medical Center, Los Angeles, California, United States of America; 4 Salix Pharmaceuticals, Inc., Raleigh, North Carolina, United States of America; University of Nevada School of Medicine, UNITED STATES

## Abstract

Diarrhea-predominant irritable bowel syndrome (IBS) is diagnosed through clinical criteria after excluding “organic” conditions, and can be precipitated by acute gastroenteritis. Cytolethal distending toxin B (CdtB) is produced by bacteria that cause acute gastroenteritis, and a post-infectious animal model demonstrates that host antibodies to CdtB cross-react with vinculin in the host gut, producing an IBS-like phenotype. Therefore, we assessed circulating anti-CdtB and anti-vinculin antibodies as biomarkers for D-IBS in human subjects. Subjects with D-IBS based on Rome criteria (n=2375) were recruited from a large-scale multicenter clinical trial for D-IBS (TARGET 3). Subjects with inflammatory bowel disease (IBD) (n=142), subjects with celiac disease (n=121), and healthy controls (n=43) were obtained for comparison. Subjects with IBD and celiac disease were recruited based on the presence of intestinal complaints and histologic confirmation of chronic inflammatory changes in the colon or small intestine. Subjects with celiac disease were also required to have an elevated tTG and biopsy. All subjects were aged between 18 and 65 years. Plasma levels of anti-CdtB and anti-vinculin antibodies were determined by ELISA, and compared between groups. Anti-CdtB titers were significantly higher in D-IBS subjects compared to IBD, healthy controls and celiac disease (P<0.001). Anti-vinculin titers were also significantly higher in IBS (P<0.001) compared to the other groups. The area-under-the-receiver operating curves (AUCs) were 0.81 and 0.62 for diagnosis of D-IBS against IBD for anti-CdtB and anti-vinculin, respectively. Both tests were less specific in differentiating IBS from celiac disease. Optimization demonstrated that for anti-CdtB (optical density≥2.80) the specificity, sensitivity and likelihood ratio were 91.6%, 43.7 and 5.2, respectively, and for anti-vinculin (OD≥1.68) were 83.8%, 32.6 and 2.0, respectively. These results confirm that anti-CdtB and anti-vinculin antibodies are elevated in D-IBS compared to non-IBS subjects. These biomarkers may be especially helpful in distinguishing D-IBS from IBD in the workup of chronic diarrhea.

## Introduction

In the clinical evaluation of chronic diarrhea, common differential diagnoses include diarrhea-predominant irritable bowel syndrome (D-IBS), inflammatory bowel disease (IBD) and celiac disease. Although the anti-tissue transglutaminase antibody (anti-tTG) has proven to be an excellent biomarker for identifying celiac disease [1], D-IBS remains a diagnosis of exclusion since the clinical criteria for IBS (Rome Criteria [2–6]) do not exclude IBD. While IBS is the most common gastrointestinal disorder with reported prevalence rates of approximately 15% of the population [7], it is considered a “functional condition” in the absence of a known “organic” biomarker.

Recently, new insights into D-IBS pathogenesis have emerged, particularly regarding the roles of acute gastroenteritis and alterations in the intestinal microbiota in the pathogenesis of this condition. D-IBS patients have alterations in their small bowel microbial flora as demonstrated by breath testing [8] as well as culture studies [9,10] and deep sequencing [11] of small bowel flora. Likewise, approximately 10% of individuals who develop acute gastroenteritis develop long-lasting D-IBS symptoms, referred to as post-infectious IBS (PI-IBS) [12–14]. Interestingly, PI-IBS may be linked to changes in the gut microbiome based on emerging animal models.

In rats, *Campylobacter jejuni* infection precipitates a phenotype similar to human PI-IBS, and leads to significant alterations in small bowel microbial colonization [15–17]. In this model, progression to an IBS-like phenotype was predicted by the presence of a bacterial toxin called cytolethal distending toxin B (CdtB). Rats infected with a mutant *C*. *jejuni* strain lacking CdtB (due to an insertional deletion mutation) exhibited significantly fewer IBS-like phenotypes compared to those infected with wild-type *C*. *jejuni* [16,18]. In rats exposed to CdtB, levels of circulating antibodies to CdtB were associated with altered gut microbial populations and reduction in interstitial cells of Cajal [19,20]. In this same work, through molecular mimicry, anti-CdtB antibodies were found to cross react with the host cell adhesion protein, vinculin. In addition, levels of circulating antibodies to CdtB and vinculin correlated with the levels of small intestinal bacterial overgrowth (SIBO) in these animals [20].

In the workup of chronic diarrhea, tTG is helpful in identifying celiac disease. Due to the lack of a specific biomarker, extensive workup is often used to separate D-IBS from IBD. Based on the pathophysiologic findings from our rat model, we assess the ability of circulating antibodies to CdtB and vinculin to differentiate D-IBS from IBD patients.

## Materials and Methods

### Subject Groups

For the validation of this new serum biomarker, subjects from a 180 center large-scale randomized controlled therapeutic trial in diarrhea-predominant IBS (D-IBS) were recruited (TARGET 3). Subjects with D-IBS were selected based on the presence of Rome III criteria [6]. Healthy controls were recruited from Cedars-Sinai Medical Center and the Beth Israel Deaconess Medical Center. All healthy controls were screened for prior history of gastrointestinal disease and for active gastrointestinal symptoms based on history and completion of a bowel symptom questionnaire. Subjects with IBD and celiac disease were recruited based on the presence of intestinal complaints and histologic confirmation of chronic inflammatory changes in the colon or small intestine consistent with Crohn’s disease, ulcerative colitis (UC) or celiac disease. In addition to histologic features, subjects with celiac disease were required to have an elevated tTG antibody and biopsy. All subjects for the study were between 18 and 65 years of age. This study was approved by the Institutional Review Board of Cedars-Sinai Medical Center and by the Institutional Review Board at the Beth Israel Deaconess Medical Center, and all subjects provided informed written consent.

Subjects were excluded from the study if they had a history of diabetes, human immunodeficiency virus (HIV), known pancreatic disease, unstable thyroid disease, and chronic narcotic use. For D-IBS subjects and healthy controls, bowel surgery (excluding cholecystectomy or appendectomy) was also criteria for exclusion.

### Patient Data

Patient demographics were obtained for all subjects including age and gender. In the case of IBD, the type of disease (UC or Crohn’s disease) was noted.

### Plasma Collection

Plasma was collected from all subjects. Specimens were collected by venipuncture in a lavender top tube, centrifuged at 3500 rpm for 15 minutes and then stored frozen at -80°C until the time of assay. In the case of the D-IBS subjects from TARGET 3, plasma was collected prior to treatment in the trial.

### ELISA testing

ELISAs were performed using complete recombinant *Campylobacter* CdtB protein (Creative Biomart, Shirley, NY) and full length human vinculin protein (Novoprotein, Short Hills, NJ) as antigens at a concentration of 1.2 μg/mL. Antigens were immobilized overnight at 4°C onto high-binding 96-well plates (Grenier Bio-One, Monroe, NC) in Borate Buffered Saline (BBS) (Medicago, Uppsala, Sweden) at a pH of 8.2. Wells were alternately coated with antigen or left uncoated in BBS to allow determination of non-specific binding of plasma. Wells were blocked with 3% bovine serum albumin in 1xPBS for 1 hour at room temperature. Coated and uncoated wells were then incubated with a 1:512 dilution of plasma for CdtB and a 1:32 dilution of plasma for vinculin for 1 hour at room temperature. Antibodies to CdtB and vinculin were used as positive controls. This was followed by 1 hour incubation with HRP conjugated secondary antibodies (Jackson ImmunoResearch, West Grove, PA). Each step was followed by a series of washes using 0.05% PBS-Tween 20. Finally, a 3,3',5,5'-Tetramethylbenzidine (TMB) substrate solution (Pierce, Rockford, IL) was used for visualization and immediately read on a BioTek Synergy HT plate reader (Winooski, VT). The optical densities (OD) were read for 90 minutes at 370nm and used to compare levels of anti-CdtB or anti-vinculin. Raw OD values were used for the data analysis.

### Statistical Analysis

Numerical variables were summarized by mean ± standard deviation. Normality of the data distributions was assessed using histograms with normal distributions curves. The anti-vinculin distribution was normalized by a square root transformation. Homogeneity of variance was assessed by Bartlett’s test.

Student’s t-test was used for comparisons of normally distributed variables between two groups. Normally distributed variables were compared across more than two groups by one-way ANOVA and Dunnett’s post hoc tests with IBS as the reference group. Categorical variables were summarized by frequency and percent. Pearson's chi-square test was used for comparison of categorical data. Receiver operating characteristic (ROC) curves were constructed in the standard fashion. Confidence intervals for areas under curves (AUC) were computed using the method of DeLong et al [21]. Sensitivity, specificity and likelihood ratios of anti-vinculin and anti-CdtB to a precision of 0.01 OD were calculated and were assessed to obtain the favorable cut-offs. The 0.05 significance level was used throughout. Statistical analysis was performed using STATA version 11.2 (STATA Corp., Texas, USA) and SAS version 9.3 (SAS Institute, Cary, North Carolina, USA).

## Results

### Patient Demographics

In total, 2681 subjects were recruited (Table 1). This included 2375 D-IBS subjects, 43 healthy subjects, 121 celiac and 142 IBD subjects (n = 73 Crohn’s, n = 69 ulcerative colitis). IBS subjects were on average 3.9 years older than the non-IBS groups (*P*<0.001). There were no differences in sex distribution of IBS and non-IBS subjects; however, percentage of females was greater in the healthy controls, IBS and celiac groups as compared with the IBD group (*P*<0.001).

**Table 1:** Patient demographics.

	Number of subjects	Age (range)	% of females
Healthy controls	43	36.0±9.9 (22–62)	67.4
D-IBS	2375	44.4±12.2 (18–65)	67.6
CD^b^	73	40.6±11.3 (18–65)	56.2
UC^c^	69	41.2±12.2 (19–63)	55.1
IBD (UC+CD)	142	40.9±11.7 (18–65)	55.6
Celiac disease	121	41.6±12.3 (19–65)	76

^a^Values are given as mean ± standard deviation

^b^ Crohn’s disease

^c^ ulcerative colitis

### Comparison of antibody titers between groups

**Fig 1:**
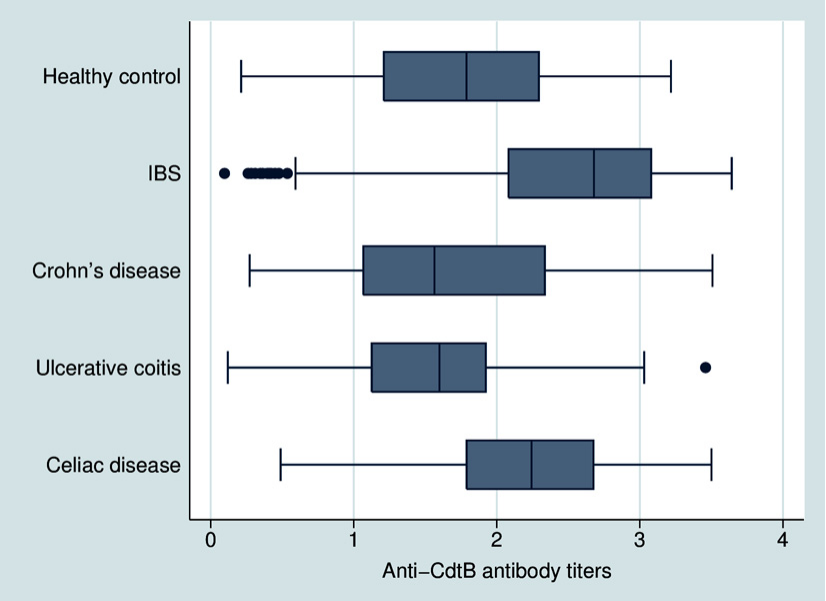
Comparison of optical density (OD) for the anti-CdtB antibody among the groups. Titers were higher in IBS subjects when compared to any other group (*P*<0.001). Titers were also higher in subjects with celiac disease when compared to healthy controls and IBD subjects (*P*<0.001). Dots represent outlier subjects beyond the whisker plot.

Using optical density levels, anti-CdtB antibody levels in D-IBS subjects (2.53±0.69) were significantly higher than healthy subjects (1.81±0.73), Crohn’s disease (1.72±0.81), ulcerative colitis (1.54±0.68) and celiac disease (2.23±0.70) (*P*<0.001) (Fig 1). There were no differences in anti-CdtB levels between healthy subjects and IBD subjects (p = 0.23); however, subjects with celiac disease had higher anti-CdtB levels than all other non-IBS groups (*P*<0.001).

**Fig 2:**
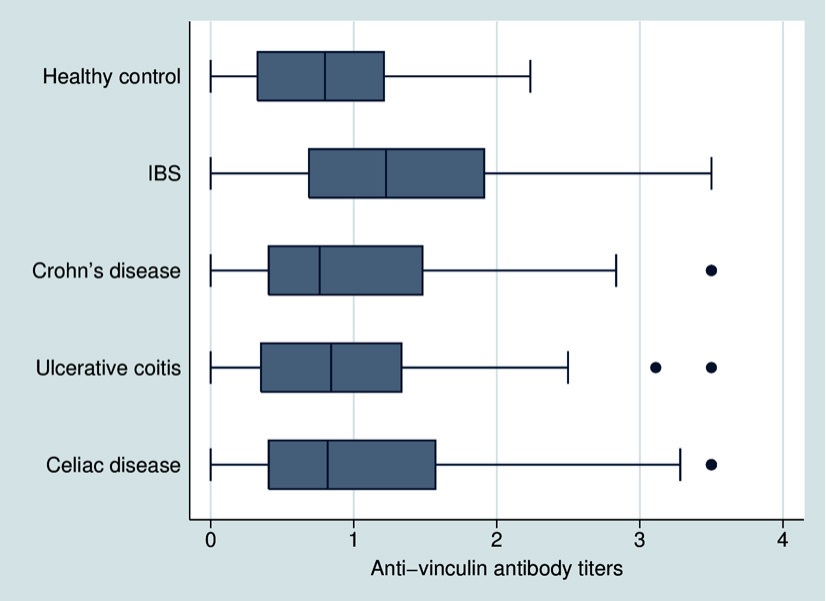
Comparison of optical density (OD) for the anti-vinculin antibody among the groups. Titers were higher in IBS subjects when compared to any other group (*P*<0.001). Dots represent outlier subjects beyond the whisker plot.

Anti-vinculin levels were also significantly higher in D-IBS subjects (1.34±0.85) when compared to healthy subjects (0.81±0.59), Crohn’s disease (1.05±0.91), ulcerative colitis (0.96±0.77) and celiac disease (1.07±0.98) (*P*<0.0001) (Fig 2). Differences in anti-vinculin levels among non-IBS subjects were not statistically significant.

### Sensitivity and specificity analyses

**Fig 3:**
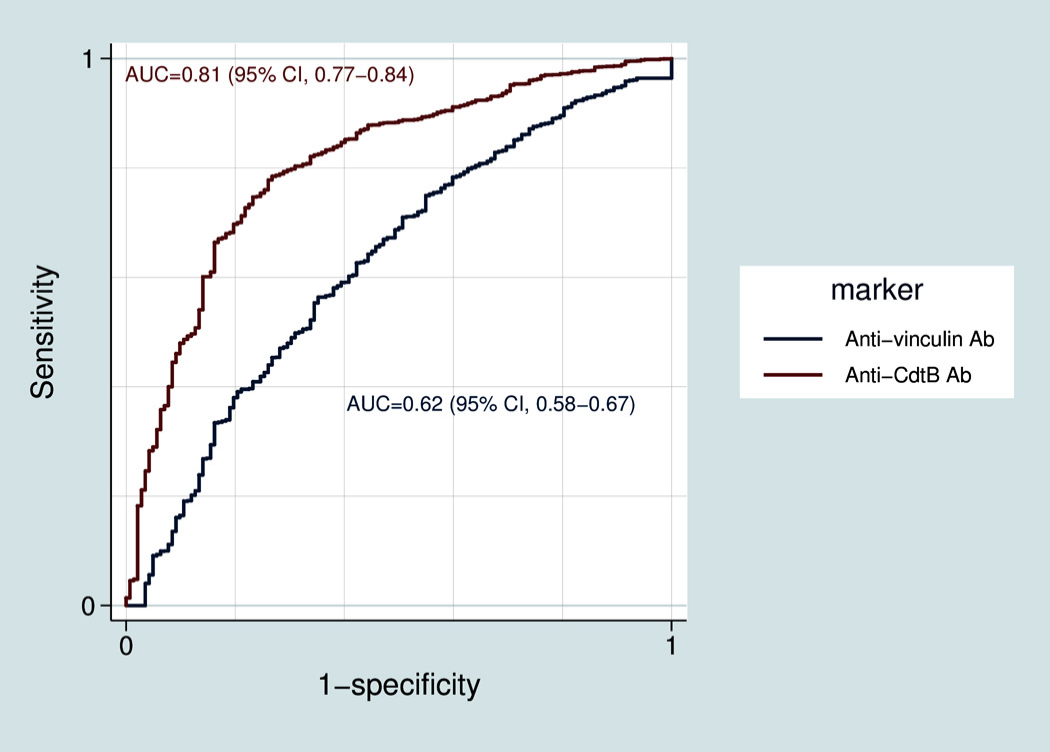
Receiver operator curve (ROC) comparing anti-CdtB and anti-vinculin levels in D-IBS subjects and IBD subjects. CI, confidence interval.

Receiver operating characteristics (ROC) were used to assess the utility of anti-vinculin and anti-CdtB levels in differentiating D-IBS subjects from IBD subjects. Fig 3 demonstrates the ROC curves for these two tests when comparing D-IBS subjects to IBD subjects. While both tests were effective at discriminating D-IBS subjects from the IBD group, the area-under-the-curve (AUC) for the diagnosis of D-IBS vs. IBD was higher for anti-CdtB than for anti-vinculin (0.81 and 0.62, respectively). In subgroup analysis, there appeared to be no difference based on the type of IBD (data not shown). The ROC curves for D-IBS compared to non-IBS, celiac subjects and healthy controls were also discriminatory (S1, S2, S3, S4 and S5 Figs).

The optical density (OD) levels for each test were then used to determine the ideal threshold for identification of D-IBS as compared to IBD. Table 2 demonstrates some potential optical density thresholds for the identification of D-IBS based on sensitivity, specificity and likelihood ratio. An ideal test would definitively diagnose D-IBS, thus reducing the need for invasive testing. Therefore, we focused on specificity and positive likelihood ratio. Based on this, the ideal threshold for anti-CdtB to identify D-IBS vs. IBD appeared to be ≥2.80, while for anti-vinculin the optimal threshold appeared to be ≥1.68. Sensitivity, specificity and likelihood ratios for the diagnosis of D-IBS vs. healthy subjects and subjects with celiac disease at different OD cutoffs were also calculated (S1 and S2 Tables).

**Table 2:** Cutoffs for anti-CdtB and anti-vinculin for the diagnosis of D-IBS vs. IBD.

OD	Specificity %	Sensitivity %	+LR^a^	-LR^b^
**CdtB**				
≥2.49	85.9	60.0	4.3	0.5
≥2.80	91.6	43.7	5.2	0.6
≥3.04	95.8	28.3	6.7	0.7
**Vinculin**				
≥1.53	80.3	37.8	1.9	0.8
≥1.68	83.8	32.6	2.0	0.8
≥1.80	84.5	28.9	1.8	0.8

^a^ positive likelihood ratio

^b^ negative likelihood ratio

## Discussion

In this study, we describe a biomarker for D-IBS based on a possible pathophysiologic mechanism of post-infectious IBS and the subsequent development of autoantibodies to vinculin in the host. The test appears specific not only for diagnosing D-IBS but in the workup of chronic diarrhea, can differentiate D-IBS subjects from those with IBD.

IBS is a condition that results in chronic changes in bowel function including diarrhea, constipation and alternating patterns. In a recent multinational initiative, IBS experts agreed that these subjects suffer from significant changes in bowel habit and bloating as principal symptoms [22]. In the absence of a clear pathophysiology of IBS, identification of subjects is based on a “diagnosis of exclusion” approach [23]. This approach involves a great deal of expense and morbidity to patients with IBS, including frequent body imaging, endoscopy and blood testing to rule out alternative organic explanations for their symptoms. While the diagnosis of celiac disease has been greatly enhanced by the measurement of serum tTG [24], there remains a need for biomarkers that distinguish IBS from IBD in the workup of chronic diarrhea.

While the rate of developing IBS after a single acute gastroenteritis is approximately 10% [12,14], military deployment data [25] and mathematical modeling [14] suggest that PI-IBS could account for a large portion of IBS in the US. PI-IBS occurs primarily, though not exclusively, after bacterial infections such as *Campylobacter jejuni* [26], *Salmonella* [27], *E*. *coli* [28] and *Shigella* [29]. One toxin commonly produced by all four of these organisms is cytolethal distending toxin, a heterotrimeric complex of three subunits, CdtA, CdtB, and CdtC, of which CdtB is the active subunit [30–32].

A validated animal model developed using *C*. *jejuni* 81–176 has been shown to exhibit an IBS-like phenotype [15]. Significantly, these rats exhibit changes in stool form, small intestinal bacterial overgrowth (SIBO) and the increased rectal intra-epithelial lymphocytes characteristic of humans with IBS [15,16]. In this model the effects appeared to be due to changes in gut neuroanatomy, with a notable reduction in interstitial cells of Cajal [16]. Further, rats infected with a mutant *C*. *jejuni* strain lacking CdtB exhibited a significantly mitigated IBS-like phenotype compared to those infected with wild-type *C*. *jejuni* [18], suggesting that CdtB was important in the development of IBS in this model. Through a series of immunologic experiments in this model, it was determined that CdtB appeared not to simply be acting through direct toxicity but rather through the cross-reaction of antibodies to CdtB with the host protein vinculin [20]. Levels of circulating antibodies to CdtB and vinculin correlated with the development and levels of SIBO in these animals [20], suggesting that circulating antibodies are linked to the reduction in intestinal vinculin.

Vinculin is a 117-kDa cytoplasmic actin-binding protein that is a key component of both focal adhesions and adherens junctions, forming the link between integrins or cadherins respectively and the actin cytoskeleton [33–36]. Furthermore, vinculin appears important in neuronal cell motility and contractility and cardiac formation, as evidenced by the neural tube, myocardial and endocardial defects in vinculin knockout mice [37], as well as stress-induced cardiomyopathy in heterozygous mutants [38]. In a recently published study, Cdt from *Helicobacter pullorum* has been shown to target vinculin in intestinal epithelial cells, triggering an atypical delocalization of vinculin from focal adhesions coupled with decreased cellular adherence [39]. Another study demonstrated that vinculin is used by the IpA toxin of *Shigella* to achieve cell entry [40].

Based on the pathophysiologic observations in this animal model of *C*. *jejuni* infection [15], we hypothesized that exposure to CdtB led to detectable immunity to CdtB and autoimmunity to vinculin based on molecular mimicry [20,41]. In this study, we evaluate whether levels of these antibodies serve as a biomarker for D-IBS in humans for the first time using a large number of IBS and non-IBS patients. We observe that plasma antibodies to vinculin and CdtB were elevated in D-IBS compared to healthy controls, subjects with celiac disease, and subjects with IBD such that the biomarkers appeared to be able to distinguish D-IBS from all non-IBS. Based on ideal cutoff titers, the test has a high specificity for identifying D-IBS compared to IBD. Since tTG is a robust test for celiac disease, in the workup of chronic diarrhea, a real unmet need is a biomarker that could reliably distinguish IBS from IBD. Interestingly anti-CdtB, but not anti-vinculin, was high in celiac disease as well. Another significant unmet need for celiac disease is a test that readily distinguishes functional symptoms from ongoing gluten exposure. Studies suggest that after gluten exposure, IBS is the second most common cause of non-responsive celiac disease, and therefore, a test that could distinguish between these causes of symptoms would be useful clinically [42].

Based on the results presented here, circulating anti-CdtB and anti-vinculin antibodies appear to be biomarkers for D-IBS and offer some unique perspectives on the pathophysiology of PI-IBS. First, these are biomarkers based on a potential mechanism for the development of IBS which may involve alterations to the enteric nervous system and gut motility. Secondly, they represent the first opportunity to make IBS a diagnosis of inclusion rather than a “diagnosis of exclusion”. Since not all D-IBS subjects test positive for these biomarkers, it is also possible that these antibodies identify a subgroup of IBS for which a mechanism and therapies could be developed. Finally, it suggests for the first time that IBS may have an organic basis. As a biomarker, measurements of anti-vinculin and anti-CdtB antibodies could help to identify D-IBS without excessive investigation and may help to target investigations in those where the test is negative.

This test may need to be put into context of existing markers used now in IBD. The most common is fecal calprotectin. As the name implies, fecal calprotectin is a stool test that identifies that inflammation is occurring in the intestinal tract. This test is used to validate the presence of IBD. The test has been used to separate IBD from D-IBS on the basis of a positive test implying IBD [43]. This paper suggests the separation of the two conditions. However, there are pitfalls with the calprotectin in that patients in remission of their IBD may be negative and essentially functions to rule in active ongoing IBD [44]. In the case of anti-CdtB and anti-vinculin, the test does the opposite. It actively diagnoses IBS. Furthermore, the title of the paper by Tibble et al [43] suggests that IBS is not an organic disease. Anti-CdtB and anti-vinculin make a further leap by suggesting that IBS might have an organic basis.

There are also some limitations to this study. First, this test has been validated for the 18–65 years age group only, and additional studies would be required to validate it in other age groups. Second, the test has a lower specificity for identifying D-IBS compared to celiac disease (see Supporting Information), although concomitant testing with anti-tTG should compensate for this. In addition, it is possible that immune responses to CdtB and vinculin could vary with differing ethnic backgrounds—Asians have been shown to have differing prevalences of certain antibodies for celiac disease [45]. Further studies are needed to address this, although the comparisons are difficult given the rarity of celiac disease in Asian populations. Lastly, it might be anticipated that 10–15% of subjects with IBD also have co-existing IBS [46]. This was not controlled for as it is difficult to identify these subjects. Previous studies have commented that symptoms compatible with IBS co-exist in patients with IBD, and are significantly more common in Crohn's disease than in ulcerative colitis patients [47], making it impossible to find a biomarker with perfect specificity or sensitivity. In the future, careful examination of IBD subjects might show that anti-vinculin and anti-CdtB antibodies identify IBS in IBD subjects also. This would be another important finding. In IBD, for example, these biomarkers might be important to suggest IBS as a cause of ongoing symptoms when there is complete mucosal healing on therapy but the patient continues to have functional symptoms.

In conclusion, this study validates the presence of anti-vinculin and anti-CdtB as blood based biomarkers that separate D-IBS from IBD and healthy controls using a large scale prospective multicenter trial. Anti-vinculin and anti-CdtB antibodies also appear part of the pathophysiology of post-infectious IBS and may identify a subgroup of D-IBS for directed therapies. Most importantly, this appears to be an important step in determining organic bases for IBS.

## Supporting Information

S1 Fig Comparison of anti-CdtB and anti-vinculin levels between D-IBS subjects and non-IBS subjects with chronic diarrhea. Receiver operator curve (ROC) comparing anti-CdtB and anti-vinculin levels in D-IBS subjects vs. all non-IBS subjects (i.e. subjects with CD, UC and celiac disease).
(TIF)

S2 Fig ROC comparing anti-CdtB and anti-vinculin levels between D-IBS subjects and subjects with celiac disease. (TIF)

S3 Fig ROC comparing anti-CdtB and anti-vinculin levels between D-IBS subjects and healthy subjects. (TIF)

S4 Fig ROC comparing anti-CdtB and anti-vinculin levels between D-IBS subjects and CD subjects. (TIF)

S5 Fig ROC comparing anti-CdtB and anti-vinculin levels between D-IBS subjects and UC subjects. (TIF)

S1 Table Sensitivity, specificity and likelihood ratios for the diagnosis of D-IBS vs. healthy controls at different OD cutoffs. (DOCX)

S2 Table Sensitivity, specificity and likelihood ratios for the diagnosis of D-IBS vs. celiac disease at different OD cutoffs. (DOCX)
