# Supplementary figures and images for: Development and Validation of a Biomarker for Diarrhea-Predominant Irritable Bowel Syndrome in Human Subjects
Source: PLoS One. 2015 May 13;10(5):e0126438. doi: 10.1371/journal.pone.0126438 (PMC4430499; doi:10.1371/journal.pone.0126438)

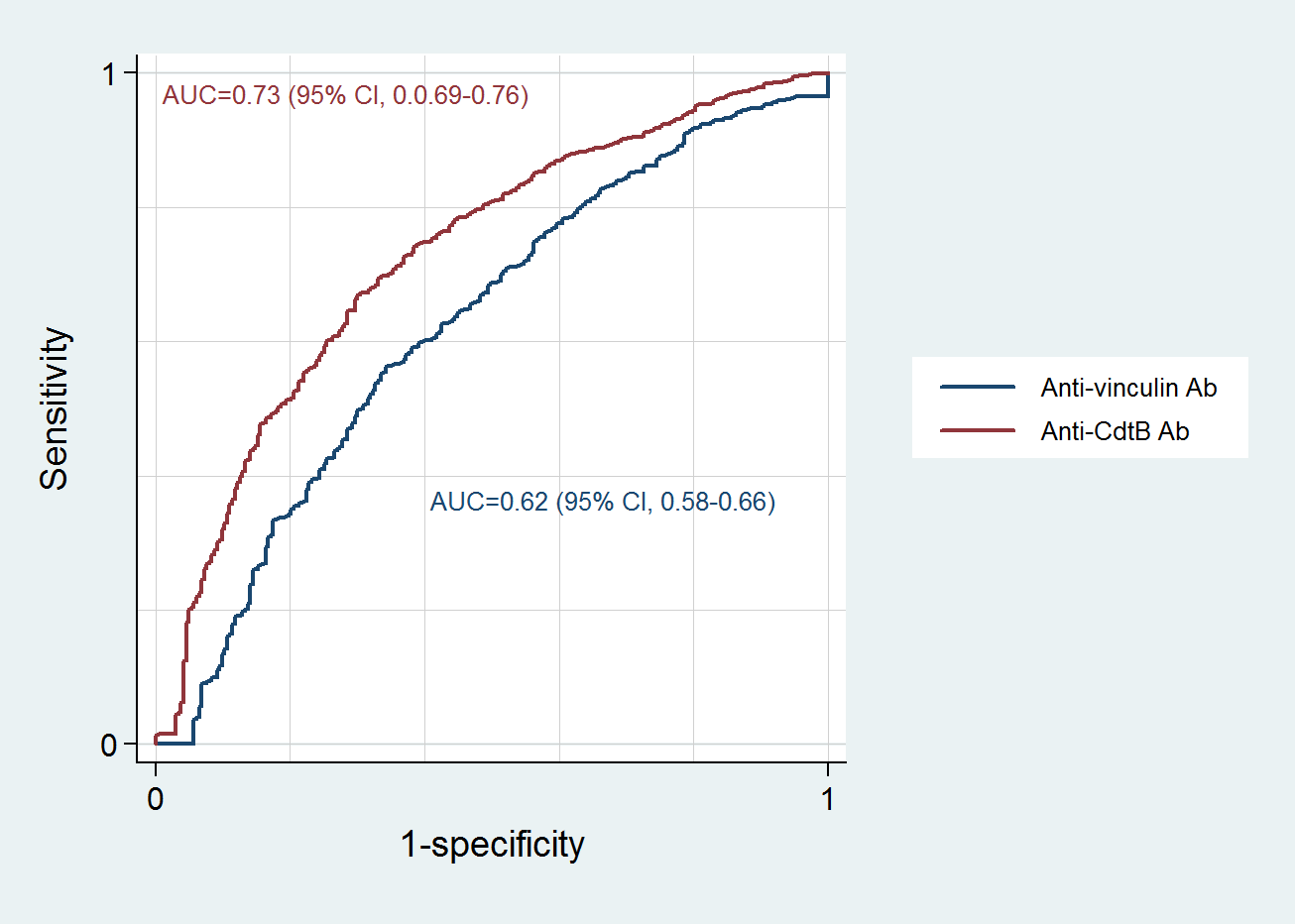

Supplement: S1 Fig — Receiver operator curve (ROC) comparing anti-CdtB and anti-vinculin levels in D-IBS subjects vs. all non-IBS subjects (i.e. subjects with CD, UC and celiac disease). (TIF) [file pone.0126438.s001.tif]

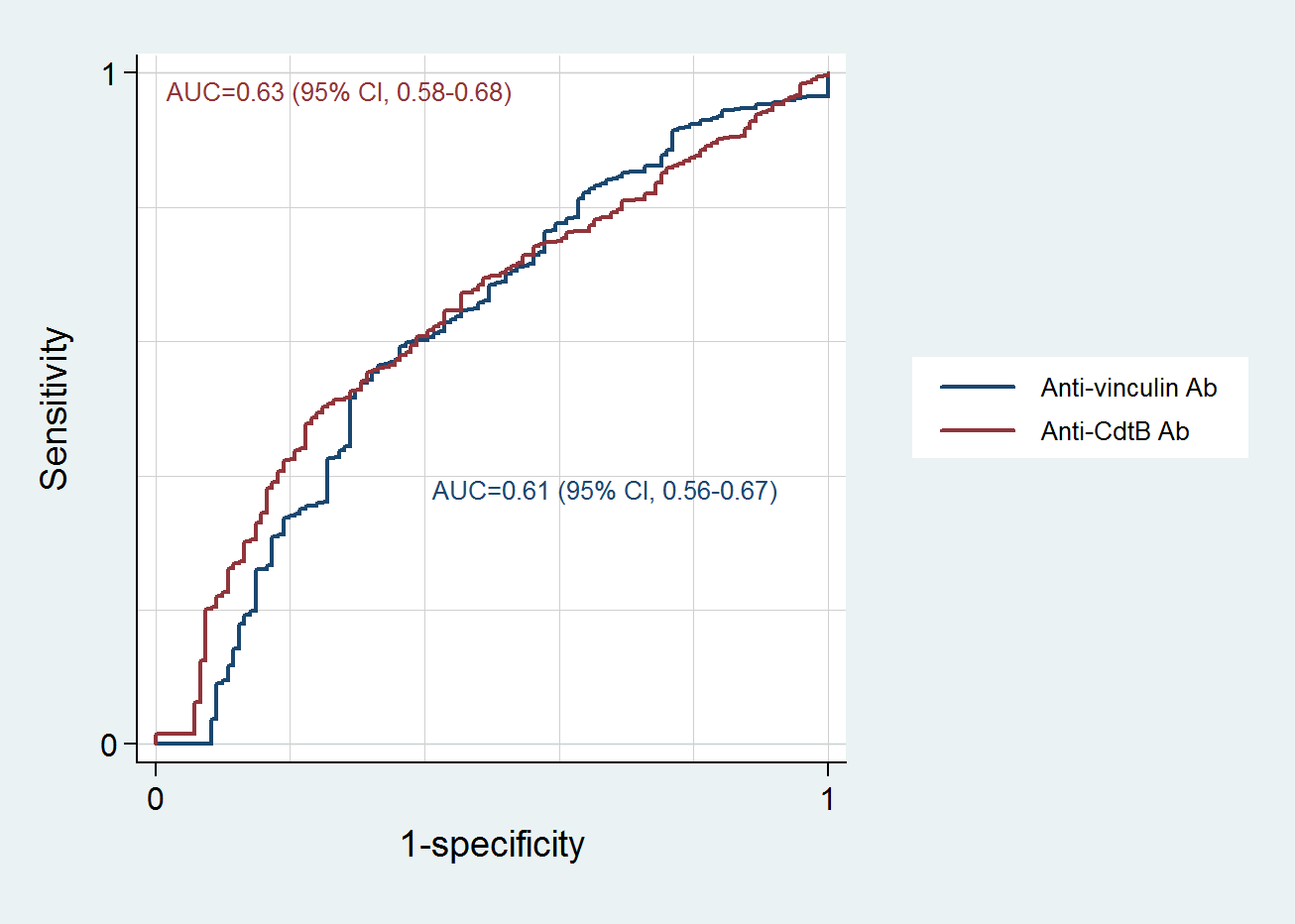

Supplement: S2 Fig — (TIF) [file pone.0126438.s002.tif]

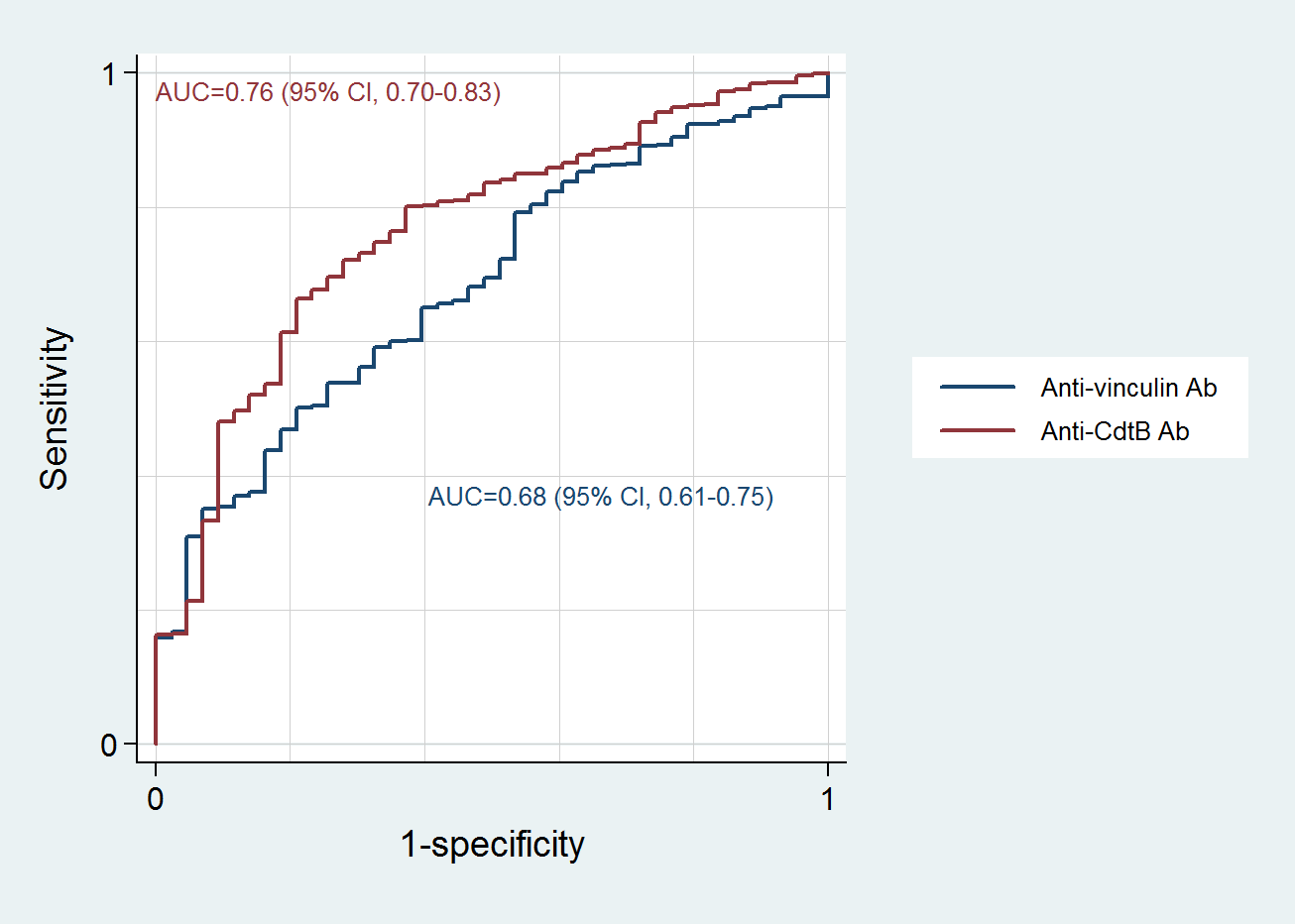

Supplement: S3 Fig — (TIF) [file pone.0126438.s003.tif]

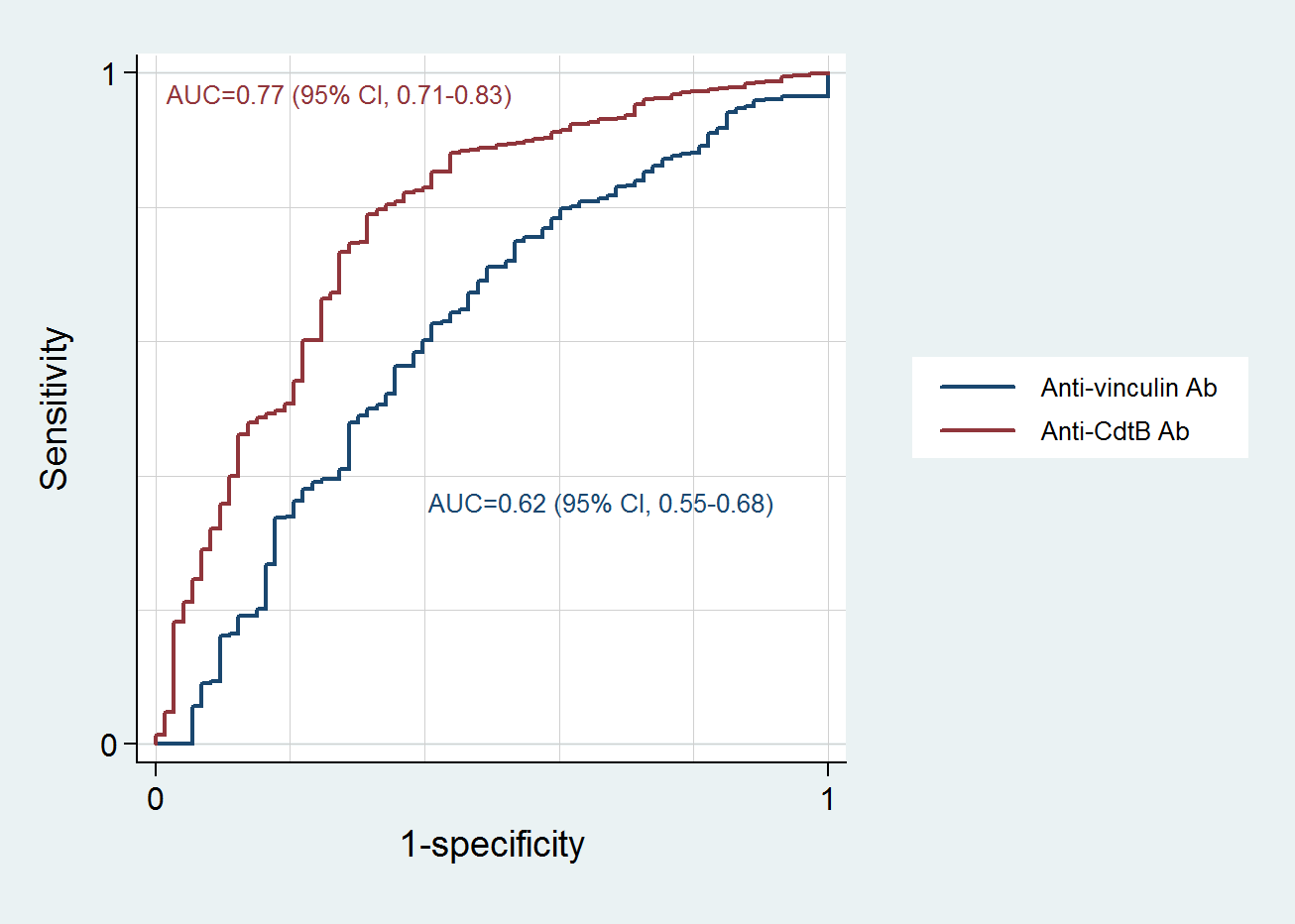

Supplement: S4 Fig — (TIF) [file pone.0126438.s004.tif]

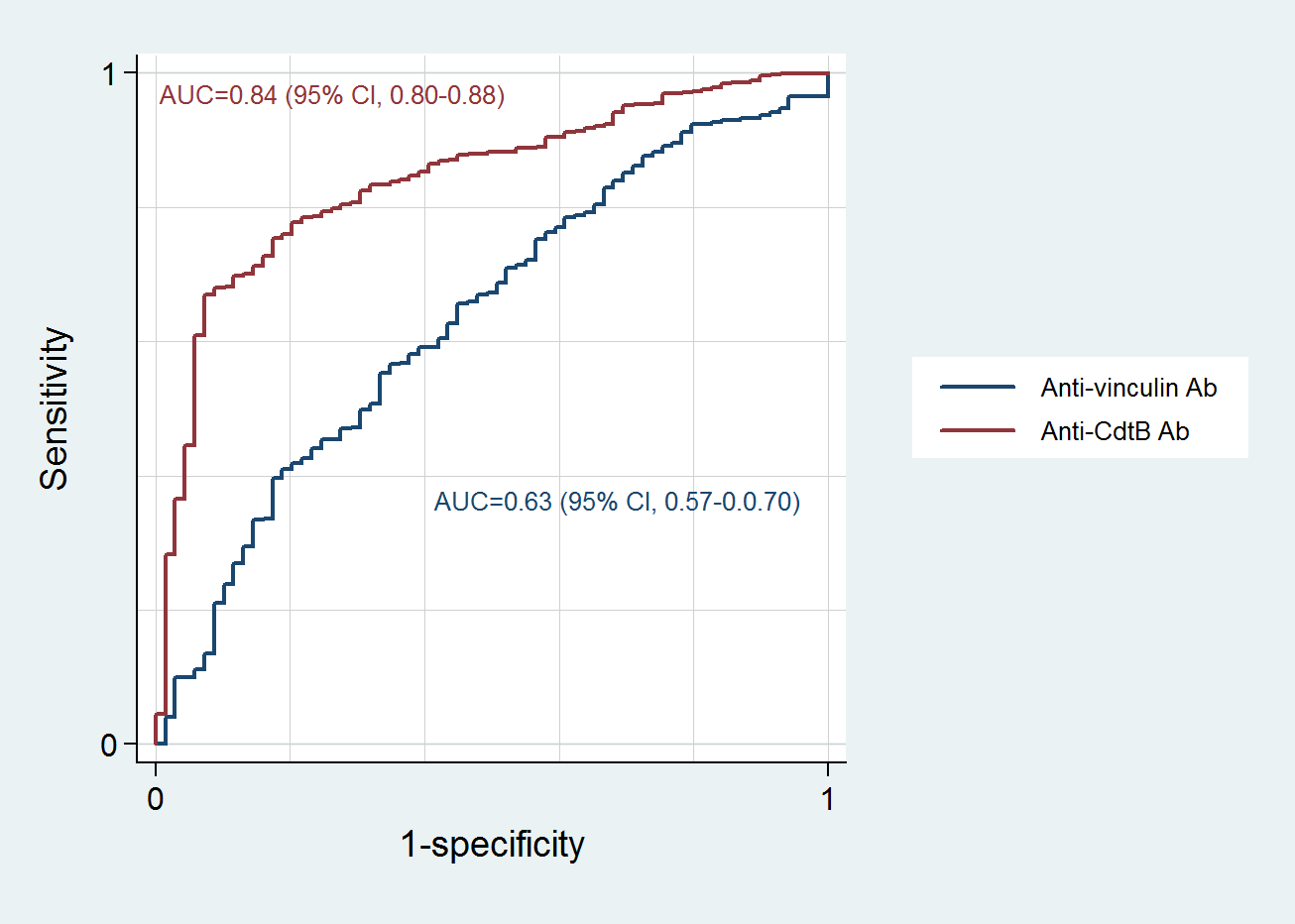

Supplement: S5 Fig — (TIF) [file pone.0126438.s005.tif]
