# Supplementary material for: Development and Validation of a Biomarker for Diarrhea-Predominant Irritable Bowel Syndrome in Human Subjects
Source: PLoS One. 2015 May 13;10(5):e0126438. doi: 10.1371/journal.pone.0126438 (PMC4430499; doi:10.1371/journal.pone.0126438)
Supplement: S1 Table — (DOCX) [file pone.0126438.s006.docx]

| **OD** | **Specificity %** | **Sensitivity %** | **+LR** | **-LR** |
| --- | --- | --- | --- | --- |
| **CdtB** |  |  |  |  |
| ≥1.53 | 83.72 | 37.60 | 2.3 | 0.7 |
| ≥1.68 | 93.02 | 32.60 | 4.7 | 0.7 |
| ≥1.80 | 95.35 | 28.93 | 6.2 | 0.7 |
| **Vinculin** |  |  |  |  |
| ≥2.49 | 81.40 | 60.00 | 3.2 | 0.5 |
| ≥2.80 | 90.70 | 43.66 | 4.7 | 0.6 |
| ≥3.04 | 93.02 | 28.29 | 4.1 | 0.8 |
