# Supplementary material for: Development and Validation of a Biomarker for Diarrhea-Predominant Irritable Bowel Syndrome in Human Subjects
Source: PLoS One. 2015 May 13;10(5):e0126438. doi: 10.1371/journal.pone.0126438 (PMC4430499; doi:10.1371/journal.pone.0126438)
Supplement: S2 Table — (DOCX) [file pone.0126438.s007.docx]

| **OD** | **Specificity %** | **Sensitivity %** | **+LR** | **-LR** |
| --- | --- | --- | --- | --- |
| **CdtB** |  |  |  |  |
| ≥1.53 | 74.38 | 37.94 | 1.5 | 0.8 |
| ≥1.68 | 80.99 | 32.59 | 1.7 | 0.8 |
| ≥1.80 | 82.64 | 28.93 | 1.7 | 0.9 |
| **Vinculin** |  |  |  |  |
| ≥2.49 | 61.16 | 60.00 | 1.5 | 0.7 |
| ≥2.80 | 79.34 | 43.66 | 2.1 | 0.7 |
| ≥3.04 | 86.78 | 28.29 | 2.1 | 0.8 |
